# Exploring the Relationship Between VMAT2 and DAT Expression, Psychotic Experiences, Craving, and Treatment Motivation in Male Patients with Methamphetamine Use Disorder

**DOI:** 10.3390/jcm13237105

**Published:** 2024-11-24

**Authors:** Mualla Keskinsezer, Ahmet Bulent Yazici, Gamze Guney Eskiler, Kaan Furkan Hamarat, Onur Davutoglu, Esra Yazici

**Affiliations:** 1 Department of Psychiatry, Faculty of Medicine, Sakarya University, 54290 Sakarya, Türkiye; mualla27@gmail.com (M.K.); onrdvtgl@gmail.com (O.D.); eyazici@sakarya.edu.tr (E.Y.); 2 Department of Medical Biology, Faculty of Medicine, Sakarya University, 54290 Sakarya, Türkiye; gamzeguney@sakarya.edu.tr; 3 Faculty of Medicine, Sakarya University, 54290 Sakarya, Türkiye; hamaratkaanfurkan@gmail.com

**Keywords:** dopamine transporter, gene expression, methamphetamine, psychic experiences, vesicular monoamine transporter

## Abstract

**Objectives:** We aimed to examine the relationship of Dopamine transporter (DAT) and vesicular monoamine transporter (VMAT-2) gene and protein levels with psychic experiences and other clinical parameters in individuals with Methamphetamine Use Disorder (MUD). **Methods:** This study included 50 males diagnosed with MUD and 50 males as a smoking control (SC) and nonsmoking control (NSC). Community Assessment of Psychic Experiences (CAPE) was administered to patients and controls; Addiction Profile Index, Treatment Motivation Questionnaire, and Substance Craving Scale were administered only to the patient group. DAT and VMAT2 gene and protein levels were determined in blood obtained from the controls and patient groups. **Results:** CAPE positive, depressive, total, and distress scores were significantly higher in the patient group. DAT protein level and *VMAT2* gene and protein levels were lower in the patient group compared to the controls. The *DAT* gene expression level was higher in the patient group compared to the controls. There was no correlation between any clinical variables and expression levels. A low *VMAT2* gene expression level could diagnose MUD with a 5% probability when NSCs were used as a reference. A high *DAT* gene expression level could diagnose tobacco use disorder (TUD) with a 99.9% probability when NSCs were used as a reference. **Conclusions:** The patient group showed more psychic experiences than healthy people. The low expression of the *VMAT2* gene was identified as a predictor of MUD, while the high expression of the *DAT* gene was predictive of TUD.

## 1. Introduction

Stimulant use disorder is a significant health problem in the world, and epidemiological data, especially on methamphetamine use, indicate that its use and use disorder have increased and become widespread in recent years [1,2].

Methamphetamine (MA) exerts its primary effects by rapidly crossing the blood–brain barrier and causing the release of catecholamines, especially dopamine, from presynaptic endings [3]. MA exerts its primary effects by inhibiting the dopamine transporter (DAT), which is involved in the removal of dopamine from the synaptic cleft, and the vesicular monoamine transporter-2 (VMAT2), which transports it to vesicles. In addition to the acute psychotic symptoms that can result from high doses of MA, prolonged exposure to MA can also lead to psychotic symptoms that persist after the substance has been eliminated [4]. Additionally, methamphetamine and certain other substances are known to significantly contribute to the onset and development of psychosis, as well as psychotic-like clinical conditions [5,6]. MA-induced psychosis is a significant concern, impacting as many as 60% of patients in certain inpatient institutions [7].

Loss of DAT function and regulation has been associated with several DA-related diseases, such as depression and schizophrenia [8]. DAT expression has also been demonstrated in human lymphocytes and has been reported to show patterns like those observed in the brain [9,10].

DAT gene expression levels in the peripheral blood and nervous system have been investigated in many neuropsychiatric disorders such as schizophrenia, Parkinson’s disease, attention deficit hyperactivity disorder, alcohol, cannabis, and nicotine addiction [11]. The results of these studies give the impression that the effects may vary according to the substance used, exposure time, and sampling [12,13,14].

Polymorphisms in the *VMAT2* (SLC18A2) gene may be associated with schizophrenia, bipolar disorder, and other neurological/psychiatric disorders [15]. VMAT2 is expressed in both the peripheral and central nervous systems, and *VMAT2* mRNA expression has been demonstrated in peripheral blood [9].

Studies have reported that similar psychotic symptoms, which are assumed to be seen only in psychiatric disorders, are seen in milder forms in a population considered to be healthy [16]. Addiction and psychotic symptoms related to chronic methamphetamine use have been linked to alterations in dopaminergic receptor density and function, especially in the mesolimbic system and striatum [17]. MA use has been reported to alter genetic expression in preclinical studies, and in one clinical study, changes in LINE-1 DNA partial methylation were associated with paranoia due to MA use [18]. However, the relationships of psychotic-like experiences and psychotic symptoms with changes in genetic expression in the context of DAT1 and VMAT2 in MA users have not been investigated.

Imaging studies conducted during the withdrawal period in human MA abusers report that VMAT2 and DAT protein levels in the caudate, putamen, and ventral striatum vary with the duration of the withdrawal period compared to control subjects [19].

Smoking is quite common in individuals with substance use disorders [20]. Although the effects of tobacco on dopamine release are well known, studies investigating the effects of smoking on DAT and VMAT expression are relatively few preclinical studies, and the results are contradictory [21].

Craving is an essential problem of withdrawal severity. Its neurobiology has been chiefly focused on neurotransmitter changes and alterations in neurotransmission, and the relationship between genetic alterations has not been sufficiently investigated [22]. Preclinical studies provide limited information regarding the role of mechanisms related to genetic expression in addiction-related brain regions in the time-dependent increase in cocaine and MA craving [23].

Dopaminergic system dysfunction in addictions is well known to reduce motivation and reinforcement. Recent preclinical studies have reported that DA-deficient mice show a complete lack of motivation in goal-directed behaviours, including feeding, although they can continue reward-related behaviours. The relationship of both *VMAT2* and *DAT1* genes with motivation has been investigated in a small number of preclinical studies [24,25].

In conclusion, while few studies examine the effect of MA use on the *DAT* gene expression profile and clinical outcomes in a clinical sample, these studies have remained at the preclinical level for VMAT2. On the other hand, to the best of our knowledge, this study is critical because it is the first study to examine MA and smoking together. Another crucial aspect of the study is that it is the first study to investigate the relationship between changes in DAT and VMAT2 at gene and protein levels and cravings, psychotic experiences, and treatment motivation.

This study has two main questions. (1) Whether the gene expression and protein levels of vesicular monoamine transporter 2 (VMAT 2) and dopamine transporter (DAT) in patients diagnosed with psychostimulant (methamphetamine) use disorder (MUD) and who regularly smoke are different from healthy controls who regularly smoke and healthy controls who do not smoke. (2) Whether there is a correlation between VMAT2 and DAT expression and sociodemographic and some clinical parameters (especially psychic experiences, negative and positive symptoms, craving, treatment motivation, and addiction severity).

## 2. Method

This study was carried out on 50 individuals between the ages of 18 and 50 who were diagnosed with MUD and tobacco use disorder (TUD) according to DSM-5 and 50 smoking controls (SCs) diagnosed with TUD and 50 nonsmoking (NSC) male individuals in the same age range who were treated at the Adult Detoxification Centre (ADC) of the Sakarya Training and Research Hospital (STRH) in Türkiye. The control group was voluntarily formed from individuals with similar demographic characteristics who live in the same region as the patients. Research indicates that substance use is predominantly observed in males, and sex-related differences can impact the transcription of monoamine transporters. Consequently, this study was designed to focus solely on male participants, thereby ensuring a homogeneous group for analysis [26,27]. Ethical approval was obtained from the Sakarya University Faculty of Medicine Clinical Research Ethics Committee with the number E-16214662-050.01.04-208337.

The sample size for the study was determined using the G*Power 3.1.9.7, a reputable statistical analysis tool. The results indicated that for evaluating DAT1 gene expression, with a targeted statistical power of 0.95 and an alpha level of 0.05 (*p* = 2.37), a minimum of 9 subjects per group would be sufficient to yield reliable outcomes. Conversely, for assessing VMAT-2 gene expression, which also aimed for a power of 0.95 and an alpha level of 0.05 (*p* = 0.80), it was found that 37 subjects would be necessary to achieve adequate statistical power. To accommodate the possibility of conducting multiple analyses during the study, the research team opted to increase the planned sample size. They established a goal of 50 subjects, incorporating a 20% increase to enhance the robustness and reliability of the findings [13,28].

This study included male participants who reported using methamphetamine within the last 36 h and were diagnosed with Methamphetamine Use Disorder (MUD) and Tobacco Use Disorder (TUD). All participants were literate and demonstrated adequate mental capacity.

Examination of medical records and participant statements revealed that individuals diagnosed with substance use disorders other than MUD or TUD had accompanying major psychiatric disorders (such as psychotic disorders, bipolar disorder, autism spectrum disorders, or eating disorders), known neurological conditions (including epilepsy, Parkinson’s disease, dementia, restless legs syndrome, or Tourette’s syndrome), or endocrinological disorders (such as diabetes or thyroid dysfunction), as well as other known hormonal disorders.

Participants who had undergone surgical procedures in the last two months, experienced head trauma, had a Body Mass Index (BMI) outside the range of 18–30, tested negative for amphetamines in urine toxicology analyses, tested positive for substances other than cannabis, or had used psychotropic drugs in the past month were also excluded from this study.

**Figure 1:**
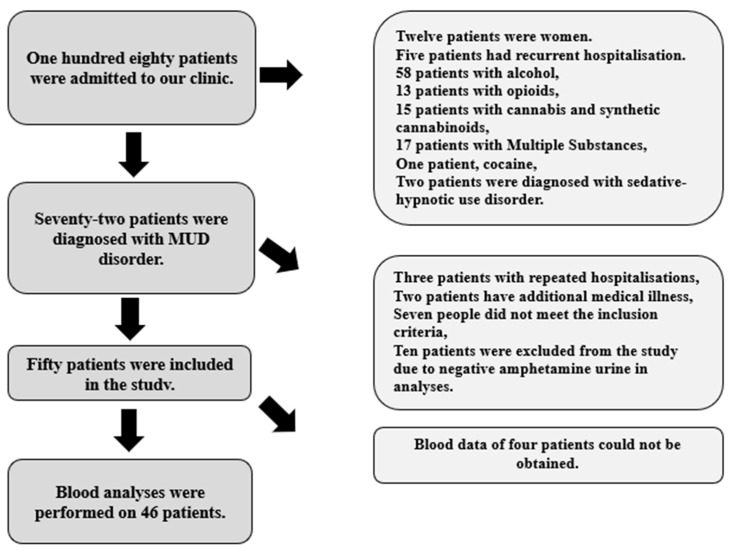
Flowchart of sample selection for patients.

A total of 180 patients were enrolled in the SEAH-ADC, and 50 of them were included in the study (Figure 1).

For the control groups, 100 healthy volunteers (50 SC, 50 NSC) who did not have a history of psychoactive substance use, had a similar male gender to the patient group, and lived in the same region participated in the study. Additionally, individuals with significant psychiatric disorders, known neurological conditions, or endocrinological disorders were excluded from the study. Those who had undergone surgical procedures in the past two months, experienced head trauma, had a Body Mass Index (BMI) outside the range of 18 to 30 in the control group, or were assessed to have limited mental capacity were also excluded. Informed consent, both written and verbal, was obtained from all participants in accordance with the Declaration of Helsinki.

For the patients, all forms and scales were administered after blood sampling on the first day of hospitalisation following the procedure described below, whereas in the control group, only SDF and CAPE were administered after blood sampling.

### 2.1. Tools

**Sociodemographic data form** (**SDF**): This form is prepared by the research team, in which clinical parameters related to substance use are evaluated in addition to the participants’ sociodemographic characteristics.

**The addiction profile index** (**API**)**:** The API is a self-assessment scale consisting of 37 questions and five different subscales. The subscales assess substance use characteristics. The validity and reliability study was conducted by Kültegin Ögel et al. in 2012. Cronbach’s alpha value was calculated as 0.89 [29].

**Community assessment of psychic experiences scale** (**CAPE**): CAPE was developed by Van Os and his team, and Stefanis and other researchers determined its validity and reliability [30]. CAPE is a 42-question self-assessment scale and can be applied to the general population. Its positive dimension assesses delusions or hallucination-like experiences, and higher scores indicate more frequent psychotic experiences. The Turkish validity and reliability study was conducted by Sevi et al. The internal consistency analysis of the Turkish version of the scale demonstrates excellent reliability with a Cronbach alpha coefficient of 0.91 [31]. The CAPE scale was included in the study because it allows for the evaluation of the sub-dimensions of psychotic experiences independently and can be utilised in both clinical and non-clinical samples.

**Treatment motivation questionnaire** (**TMQ**)**:** TMQ is a five-point Likert-type structured scale designed to measure the level of treatment acceptance and participation in treatment of individuals diagnosed with the disease, and it is a widely utilised tool in addiction research. It was developed by Ryan et al. in 1995, and its validity and reliability study in Turkey was conducted in 2006 by Evren et al. The Cronbach’s alpha value of the Turkish version of the scale was calculated as 0.84 [32,33].

**Substance craving scale** (**SCS**)**:** The SCS was adapted from the Penn Alcohol Craving Scale for individuals with a non-alcoholic substance use disorder. It assesses the desire to use substances over the past week in terms of duration, frequency, intensity, craving, and resistance. The Turkish version of the scale has been demonstrated to be both valid and reliable, and it has been effectively utilised in numerous studies on addiction [34]. The Cronbach’s alpha value of the scale was calculated as 0.84 [35].

### 2.2. Laboratory Methods

**Collection and analysis of blood samples:** One EDTA tube (for RNA isolation) and one vacuum gel tube (for serum collection) containing 5 mL of venous blood samples were collected between 09.00 and 09.30 in the morning after a twelve-hour overnight fast. The EDTA tubes were gently inverted after blood collection and stored at −80 °C until RNA isolation was performed. Immediately after the blood samples were taken into biochemistry tubes for serum collection, they were centrifuged at 1500 g for 10 min; the separated serum portion was placed in Eppendorf tubes, coded by number, and stored at −80 °C until ELISA analyses were performed. When all samples were completed, the samples were removed from −80 °C and kept at room temperature.

**RT-PCR analysis:** Total RNA isolation was performed from the collected whole blood samples using TRIzol reagent (Thermo Scientific, Waltham, MA, USA) according to the appropriate kit protocol. The concentrations of isolated total RNAs were measured using a Qubit 4.0 Fluorometer (Invitrogen, Waltham, MA, USA). According to the kit protocol, isolated total RNAs were translated into cDNA using a High-Capacity cDNA Reverse Transcription Kit (Thermo Fisher Scientific, Waltham, MA, USA). Changes in *VMAT2* (*SLC18A2)* and *DAT* (*SLC6A3)* mRNA levels were determined by StepOnePlus Real Time-PCR (Applied Biosystems, USA) using the synthesised cDNAs for RT-PCR analysis. TaqMan^®^ Gene Expression Assays (Thermo Fisher Scientific, USA) primers (Hs00997374-SLC6A3, Hs00996835-SLC18A2) and the corresponding TaqMan^®^ Gene Expression Master Mix (Thermo Fisher Scientific, USA) were used for the relevant genes and the reference gene ACTB (β-Actin), and then the reaction was performed by adjusting the PCR conditions and the number of cycles. The results were analysed using a data analysis software (REST (2009 V2.0.13)), and changes in the expression levels of genes were determined as fold changes.

**ELISA analysis:** Changes in the levels of VMAT2 and DAT protein in THE serum samples were analysed by using a Human Vesicular Monoamine Transporter 2 (VMAT2) ELISA Kit (E7707Hu, BT-LAB, Shanghai, China) and Human Dopamine Transporter (DAT1) ELISA Kit (E4541Hu, BT-LAB, China), according to the kit protocol recommended by the company, and measured at 450 nm wavelength with an ELISA Plate Reader (Hangzhou Allsheng, Hangzhou, China). The data obtained were analysed using Curve Expert software version 1.4. The absorbance values of the standards were entered, and the amount of the related protein was calculated using the formula y = ax + b for each absorbance value. The VMAT2 and DAT protein levels were expressed as ng/mL.

### 2.3. Statistical Analysis

Statistical analyses were performed using an IBM SPSS Statistics 22.0 programme. Normality assumption was checked with kurtosis and skewness values [36]. A one-way ANOVA was employed for continuous variables that exhibited a normal distribution, whereas a Kruskal–Wallis test was utilised for those that did not. Subsequent post hoc analyses were conducted using a Tukey or Tamhane’s T2 tests, as appropriate. A chi-square or ‘Fisher exact’ test and post hoc analyses were used for categorical variables [37]. Correlation analyses (Pearson for normally distributed data and Spearman for non-normally distributed data) were used. A COCOR analysis was used to compare the correlations of the groups [38] statistically. The correlation analyses conducted in this study did not indicate a statistically significant relationship among the variables, particularly concerning the primary objectives of the research. Considering considerable differences observed in gene and protein expression levels across the various groups, a multinomial regression analysis was undertaken. The Odds Ratio (OR = Exp(B)) was used to assess effect size. Confidence levels were 95% and *p* < 0.05 for pairwise comparisons and 95% and *p* < 0.017 for triple comparisons with Bonferroni correction.

## 3. Results

### 3.1. Comparison of Patient and Control Groups in Terms of Sociodemographic Data and Clinical Characteristics

All patients and healthy control groups included in our study were male, and no statistically significant difference was found in mean age and BMI (*p* > 0.017). A statistically significant difference was found when the three groups were compared in terms of age at initiation of smoking and amount of daily use, occupation, educational status, marital status, and level of active alcohol consumption (*p* < 0.05).

Sociodemographic data and clinical characteristics of the patient and control groups were presented in Table 1.

### 3.2. Substance Use Characteristics of Patient

The mean age of the patients when they started using illicit substances for the first time was 19.16 ± 7.00 (min:10−max:40). Mean duration of MA use of patients was 2.5 ± 1.92 years (min:1–max:12). According to the frequency of methamphetamine use, 38 (76%) patients reported using methamphetamine every day, while 12 (24%) patients reported using it at least twice a week. While 33 of the patients were hospitalised for the first time, 17 of them had repeated hospitalisations.

### 3.3. Comparison the Community Assessment of Psychic Experiences Scale Scores Between Patient and Control Groups

When the CAPE scores were compared, it was found that there was a statistically significant difference between the patient and control groups in the positive and depressive psychic experiences subscales and total psychic experiences and distress scores (*p* < 0.017) (Table 2). In the post hoc analysis, it was determined that the patient group had significantly higher scale scores than both groups (*p* < 0.05), while there was no statistically significant difference between the control groups (*p* > 0.05)

### 3.4. Comparison of Gene Expression and Protein Levels of Groups

When the patient and control groups were compared in terms of DAT and VMAT2 gene expression levels, a significant difference was found between them (*p* < 0.017) (Table 3). In the post hoc analysis, the DAT gene expression level was found to be lower in the patient group, compared to the SC, and higher in the NSC (*p* < 0.05), while it was found to be significantly higher in the SC compared to both groups (*p* < 0.05). The VMAT2 gene expression level was found to be significantly lower in the patient group compared to both control groups (*p* < 0.05), and no significant difference was found between the smoking and nonsmoking control groups (*p* > 0.05).

**Figure 2:**
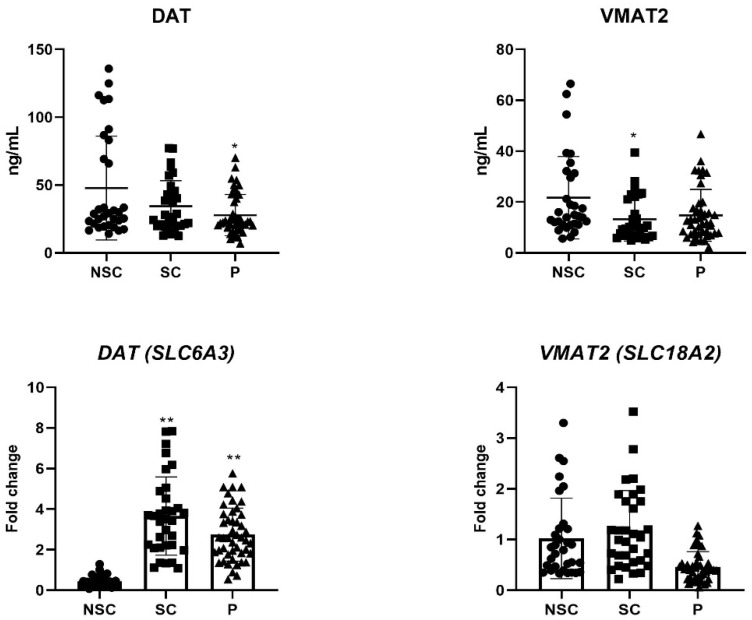
Gene and protein levels of the groups [DAT (*p* = 0.015), VMAT2 (*p* = 0.044), *DAT* (*p* = 0.000), *VMAT2* (*p* = 0.000)] * *p* < 0.05, ** *p* < 0.001, P; Patient Group, SC; Smoking Control Group, NSC; Non-smoking Control Group.

When the patient and control groups were compared in terms of DAT and VMAT2 protein levels, a significant difference was found in the DAT protein levels (*p* < 0.017). However, no significant difference was found in VMAT2 protein levels (*p* > 0.017) (Table 3). In the post hoc analysis, the DAT protein levels of the patient group were found to be significantly lower than the NSC group (*p* < 0.05), while no significant difference was found between the patient group and the SC group and between the SC and the NSC (*p* > 0.05). The gene expression and protein levels of the groups are presented in Figure 2.

### 3.5. The Relationship Between Gene Expression and Protein Levels and Sociodemographic and Clinical Data in Patient and Control Groups

In the patient group, the DAT and VMAT2 protein levels showed a significant positive correlation (*n* = 44 r = 0.721, *p* < 0.001). No significant correlation was found between other gene expression levels and protein levels (*p* > 0.05)

No significant correlation was found between patient group gene expression and protein levels and age; BMI, age, and duration of smoking initiation; age and duration of substance initiation; monthly income; physical activity; education level; and caffeine consumption (*p* > 0.05).

There was no statistically significant relationship between gene expression and protein levels and addiction profile, treatment motivation, and craving scores in the patient group (*p* > 0.05)

### 3.6. Correlation of Gene Expression and Protein Levels Among Themselves and Between Psychic Experiences Scale Scores in Control Groups

Like the patient group, a highly significant positive correlation was found between the DAT and VMAT2 protein levels in both control groups (SCG; *n* = 30 r = 0.786 *p* < 0.001, NSCG; 31 r = 0.936 *p* < 0.001). No significant correlation was found between other gene expression levels and protein levels (*p* > 0.05)

When the correlation of gene expression and protein levels with sociodemographic data in the control groups was examined, no significant correlation was found with age, BMI, age of smoking initiation, and education level. In contrast, a negative correlation was found between DAT and VMAT2 gene expression levels and caffeine consumption in NTSCs. A positive correlation was found between monthly income and DAT and VMAT protein levels (Table 4).

There was no statistically significant correlation between gene expression and protein levels and psychic experiences total and subscale scores in the control groups (*p* > 0.05).

### 3.7. Statistical Comparison Analysis of Gene Expression and Protein Level Correlations of Patients and Both Control Groups (COCOR)

Comparison of the correlations of DAT and VMAT2 protein levels between the patient and nonsmoking control group showed that the difference between the correlations of these two groups was significant (z = −3.276 *p* = 0.001). However, no other correlation data were found to be significant among other comparisons.

### 3.8. Regression Analysis

We used a multinomial logistic regression model to analyse whether gene expression and protein levels in blood are predictive of a person’s methamphetamine or tobacco use.

The model we wanted to create met the assumptions of multinomial logistic regression to have a nominal dependent variable with at least three categories (P, SC and NSC), no significant outliers in the data set, and no strong multicollinearity between independent variables.

A multinomial logistic regression model, including four independent variables, including DAT gene and protein levels and VMAT2 gene and protein levels, was established with reference to the SC group.

In the multinomial logistic regression analysis applied to determine the effects of DAT and VMAT2 gene and protein levels on the substance and smoking characteristics of individuals (P, SC and NSC groups), the resulting regression model was statistically significant (Likelihood Ratio Test X2 = 145.524, Sd = 8, *p* < 0.001) (Goodness-of-Fit Pearson X2 = 74.526, *p* = 1.000).

The independent variables of the study, gene expression and protein levels, predicted 87% of the variance (Pseudo R2-Nagelkerke = 0.873). When the classification table was analysed, the regression model correctly classified the group diagnosed with methamphetamine use disorder by 82.5%, the SC group by 72.4%, and the NSC group by 96.6%. The overall accuracy rate for classification was 83.7%.

The multinomial logistic regression model with patient and control groups was given in Table 5 and Table 6.

According to Exp (B) values in the comparison of the groups where the SC group was taken as the reference category, a person with a high DAT protein level had a 48% probability of being diagnosed with MUD (patient), and the probability of being diagnosed with MUD was 6% (1.07 times) lower than the probability of being a healthy smoker. A person with a low *SLC6A3 (DAT)* gene expression level had a 36% chance of being diagnosed with MUD and was 41% (1.7 times) less likely to be diagnosed with MUD than a SC. A person with an elevated VMAT2 protein level had a 53% chance of being diagnosed with MUD and was 1.12 times more likely to be diagnosed with MUD than an SC. A person with a high *SLC18A2 (VMAT2)* gene expression level had a 5% chance of being diagnosed with MUD, and the probability of being diagnosed with MUD was 94% (18 times) lower than the probability of being a SC (Table 5).

In the model where the NSC group was taken as reference, according to the SC group’s Exp (B) values, a person with a high DAT protein level had a 52% probability of belonging to the SC group. The probability of belonging to the SC group is 1.11 times higher than the probability of being an individual belonging to NSC group. A person with a high *SLC6A3 (DAT)* gene expression level could belong to the SC group with a 99.99% probability, and the probability of belonging to the SC group was 310.352 times higher than the probability of being a healthy smoker. A person with a high VMAT-2 protein level can belong to the SC group with a 43% probability, and the probability of belonging to SCG is 24% (1.32) times lower than the probability of belonging to the NSC group. A person with a high *SLC18A2 (VMAT2)* gene expression level could belong to the SC group with a 0.13% probability, and the probability of belonging to the SC group was 98% (71 times) lower than the probability of belonging to the NSC group.

## 4. Discussion

The main findings of this study were that people with MUD had more psychic experiences than healthy people. The DAT and VMAT2 gene and protein levels measured from peripheral blood showed a statistical difference between the groups. There was no correlation between gene and protein levels and clinical parameters. In further analysis, the *VMAT2* gene expression levels were predictive for methamphetamine use disorder, and the *DAT* gene expression levels were predictive for smoking.

The gender of all patients and control groups who participated in our study was male. It was reported in the TUBIM 2021 report that 92.9% of methamphetamine users in Turkey are male [39]. Since inpatients in addiction treatment centres are predominantly male and gene expression and protein levels may vary depending on gender, the patient and control groups were determined as male gender. [40].

The groups showed no significant differences in mean age or body mass index (BMI). However, significant differences were observed in the age of smoking initiation, daily smoking habits, occupation, education level, marital status, and active alcohol consumption. Substance use disorder is generally associated with lower socio-economic and educational levels. People with substance use disorder consume more alcohol-like substances and start using addictive substances, including nicotine, at an earlier age [41,42]

Our study found that individuals with MUDs reported significantly more psychic experiences, except negative experiences, compared to non-users. In a study examining the correlation between substance use and psychotic experiences, it was discovered that nearly fifty percent of individuals with a history of substance use reported enduring psychotic experiences, with a particularly strong association identified with the use of amphetamines [43]. Furthermore, a recent meta-analysis focused on a younger demographic revealed that individuals who engage in substance use are approximately twice as likely to report experiencing symptoms resembling psychosis [44]. In the context of psychotic-like experiences, it is essential to consider not only the impact of MA use on the dopaminergic system but also the complex bidirectional relationship between substance use and psychosis [45]. Furthermore, factors such as low socioeconomic status, health inequality and negative societal attitudes must be considered to fully understand the development of these experiences [46]. However, neurocognitive functions were not evaluated in this study, which is an important limitation to consider when assessing patients with low functioning and high levels of psychotic experiences. Cognitive functioning and neuropsychological performance, in conjunction with ethnic background and the concurrent use of other psychoactive substances, significantly influence the manifestation of psychotic symptoms, as well as expressions of violence and hostility. These factors represent crucial functional dimensions for individuals within societal contexts [47,48].

The current study found no statistically significant difference in psychic experiences and subscale scores between the SC and NSC groups. Some publications have reported a direct relationship between smoking and psychic experiences [49,50]. However, the absence of a significant difference in our findings may be attributed to several factors, including variations in sample size, selection methods, levels of smoking, and measurement techniques employed. In a study involving a sample of 1680 randomly selected individuals, Bhavsar et al. established a relationship between the dosage of smoking and the incidence of psychotic experiences, as assessed by the Psychosis Screening Questionnaire [51].

DAT protein levels were found to be the highest in the nonsmoking healthy control group, intermediate in the smoking control group, and the lowest in the patient group in accordance with the literature. In the between-group comparison (post hoc test), DAT protein levels were found to be significantly lower only in the patients compared to the NSC group. Several alterations, such as changes in DAT and VMAT activity, decreases in DAT levels, and tyrosine hydroxylase (TH) activity due to overstimulation of the dopamine system, have been found in methamphetamine users [52]. Chronic exposure to nicotine and smoke has been shown to reduce expression levels, particularly in the striatal regions [53,54,55]. This information can help interpret the results of the current study. It suggests that smoking decreases the availability of dopamine transporters (DAT), like the effects of methamphetamine (MA) use. However, MA use appears to have an additional compounding effect, further decreasing DAT levels.

Our study found a significant difference between the three groups and in pairwise comparisons in terms of *DAT* gene expression level. In the *p* group, a high expression level was found compared to the NSC group, and a low expression level was found compared to the SC group.

There are preclinical and clinical studies investigating the effects of psychostimulants and other drugs on gene expression in peripheral blood and CNS [56]. Postmortem studies in chronic cocaine users reported decreasing DAT gene expression in the midbrain [57,58]. Another study reported decreases in *DAT* gene expression and dopamine levels after the administration of cocaine in cell culture [59]. Studies in human cell cultures have reported that short-term exposure to stimulants can cause transient down-regulation (amphetamines) or up-regulation (cocaine) of *DAT* expression. The difference between cocaine and amphetamines has been explained as a compensatory mechanism to reduce the intracellular toxic effects of amphetamines due to their effects via VMAT2 [12]. The literature on *DAT* gene expression includes acute and chronic applications and different substances and contains contradictory results.

Since nicotine does not bind to a site on the DAT protein, it is not a competitor or substrate for DAT [60]. However, acute systemic nicotine administration caused an increase in DA uptake, an increase in DAT cell surface expression, and a decrease in intracellular DAT protein [61]. In one study, the timing of the effect of smoking on DAT gene expression is important, and the impact of the last dose taken varies in a matter of minutes [62]. In our study, while the *p* group was conducted in clinical conditions where smoking was more controllable, the lack of similar restrictions for the SC group may have prevented us from excluding the temporal effect of nicotine. Secondly, MA deprivation in the *p* group may have altered *DAT* gene expression levels. The other question to be answered is about the effects of MA and nicotine together on DAT gene expression. Unfortunately, the literature does not have enough information to answer these questions. New and comprehensive studies on this subject are needed.

Our study found no statistically significant difference between the groups regarding VMAT2 protein level. Autopsy studies report that in MA users, VMAT2 levels are not significantly reduced except in two cases in the nucleus accumbens and putamen [63]. A study with mice showed that VMAT2 function in dopamine neurons was suppressed by up to 75% for 24 h after MA exposure, and the function was impaired for up to approximately one week [64,65].

VMAT2 protein was found to increase in the striatum of nicotine-treated mice in a time-dependent manner, and *VMAT2* mRNA was found to be elevated in the substantia nigra pars coma and ventral tegmental area. This indicates gene expression and subsequent protein synthesis. VMAT2 increase may be a compensatory mechanism to restore and maintain synaptic transmission in dopaminergic midbrain neurones during nicotine withdrawal [66].

In our study, the *VMAT2* gene expression level was significantly lower in the *p* group compared to healthy controls, while no significant difference was found between the SCs and NSCs.

Animal experiments have reported that MA exposure leads to a decrease in synaptic and vesicular VMAT2 expression levels. [67,68,69] Both in vitro and in vivo studies have reported that the increase in VMAT2 expression acts as a neuroprotective mechanism in dopamine neurons [68]. It has been reported that the dopaminergic pathway and neurons in the midbrain are protected, and damage is reduced after MA exposure in mice with increased VMAT2 expression [70]. Studies have shown that increasing functional VMAT2 expression in dopamine neurons can affect the neurotoxicity and potentially addictive properties of MA [71]. The discovery of new neuroprotective agents and clinical applications may lead to new approaches in treating MA, indicating the need for more comprehensive studies.

The majority of studies examining the relationship between DAT and VMAT2 levels and MA use are preclinical and postmortem in nature, and the number of such studies is limited. This study represents a significant contribution to the literature on this subject, as it is the first to examine DAT and VMAT2 levels in peripheral blood in chronic MA and tobacco users, together with clinical parameters. The second primary objective of this study was to investigate the relationship between VMAT2 and DAT levels and psychic experiences. However, no significant correlation was identified between gene and protein levels and the scales in the patient and control groups. This may be attributed to the psychic experiences scale being a self-report scale or the scale not being sufficiently sensitive to epigenetic changes measured in peripheral blood.

No significant correlation was detected between gene expression and protein levels with other scales performed in the patient group. Studies have shown that epigenetic changes that occur with substance abuse are sensitive to temporal relationships [72]. Determining the relationships between epigenetic changes and long-term clinical parameters in experiments conducted with clinical samples under natural conditions brings many difficulties. In our findings, the study design should be structured with electrophysiological parameters that can be associated with epigenetic changes. The need for a larger sample size for association studies can be presented as another explanation.

In our study, multinomial logistic regression analysis between groups found that gene expression and protein levels predicted whether the individual was a chronic methamphetamine (plus tobacco) or tobacco user with a rate of 87%, and, in particular,, gene expression levels could be a guide in these issues. According to the results of comparing the group with SC and the group with MUD, low *VMAT2* gene expression levels could predict with high accuracy that the person is likely to be in the group with MUD. Another finding is that the *DAT* gene expression level could be a highly accurate predictor in distinguishing smoking controls from nonsmokers.

This study had limitations. Firstly, the fact that the DAT and VMAT2 levels were measured in peripheral blood samples rather than CSF means that we need to know to what extent the data we obtained are indicative of CNS levels. The single measurement in this study may cause the effect of the change in expression levels to be missed over time. The lack of evaluation of accompanying mental disorders using either clinical or non-clinical scales in both the control and patient groups may be considered a significant oversight. Additionally, the biochemical analyses conducted on both groups were incomplete. The loss of data in both the patient and control groups, the exclusion of female participants, a small sample size, and the broad age range all reduce the generalizability of the findings. The fact that the exact doses of methamphetamine used by the patients were not known led to limitations in the interpretation of the results.

This study had notable strengths. All patient and control groups were assessed for psychic experiences. In addition, the evaluation of many clinical parameters in our research is essential for understanding the effect of gene expression and protein levels. The fact that our study was conducted in an inpatient clinic, that all subjects were male and smokers, that the control groups were formed as two groups to exclude the effect of smoking, and that all patients were urine amphetamine positive are other essential strengths. Our study will contribute to the literature, as it is the first in many subjects.

## 5. Conclusions

The results of this study supported the previous literature reporting that individuals with MUD have more positive, depressive, and general psychic experiences than healthy individuals. The DAT and VMAT2 gene and protein levels were significantly different in individuals with MUD compared to healthy individuals. The differences in gene and protein levels between the nonsmoking control group and the smoking control group suggest that smoking is a confounding factor. On the other hand, when all variables were included, gene expression levels came to the fore as a predictive factor. No significant correlation was found between gene expression, protein levels, psychic experience scores, and clinical parameters. In addition to the remarkable results of the study, to the best of our knowledge, this is the first study to investigate the relationship between DAT and VMAT2 gene and protein levels and psychic experiences and other clinical parameters in people with MUD and smoking disorder. Further studies with larger samples and more sensitive methods for clinical parameters are needed.

## Figures and Tables

**Table 1:** Comparison of sociodemographic and clinical characteristics of the patient and control groups.

Variable		P (*n* = 50)		SC (*n* = 50)		NSC (*n* = 50)			*p*
		Mean ± SD		Mean ± SD		Mean ± SD			
Age		30.68 ± 7.30		30.66 ± 7.28		30.24 ± 7.90			0.947
BMI		24.43 ± 4.53		26.07 ± 3.95		26.07 ± 3.95			0.026
First Smoking Age		14.70 ± 3.88		18.22 ± 5.47		19.06 ± 6.05			<0.001
		***n***	**%**	***n***	**%**	***n***	**%**	χ^2^=	*p*
Occupation	Working	23	46	43	86	31	62	17.74	<0.001
	Not Working	27	54	7	14	19	38		
Profession	Does not have	18	36	7	14	18	36	63.88	<0.001
	Officer	0	0	11	22	15	30		
	Worker	9	18	29	58	16	32		
	Indefinite	23	46	3	6	1	2		
Education Level	Primary	5	10	0	0	2	4	56.08	<0.001
	Secondary	26	52	3	6	1	2		
	High	15	30	37	74	39	78		
	University	4	8	10	20	8	16		
Marital Status	Married	19	38	24	48	16	32	23.30	<0.001
	Single	19	38	24	48	34	68		
	Divorced	12	24	2	4	0	0		
He lives with	Family	47	94	35	70	34	68	18.26	<0.001
	Friends	0	0	3	6	9	18		
	Alone	3	6	12	24	7	14		
Place of residence	Rural	17	34	1	2	3	6	25.51	<0.001
	Urban	33	66	49	98	47	94		
Family Structure	Nuclear	33	66	35	70	41	82	3.491	0.175
	Extended	17	34	15	30	9	18		
Monthly Income	<1 MW	27	54	2	4	9	18	36.91	<0.001
	1–2 MW	14	28	36	72	32	64		
	>2 MW	9	18	12	24	9	18		
Caffeine Consumption	Yes	29	58	35	70	36	72	2.139	0.343
	No	21	42	15	30	14	28		
Daily Smoking	<1 *p*	8	16	21	42			9.29	0.017
	1 *p*	29	58	22	44				
	1–2 *p*	11	22	7	14				
	>2 *p*	2	4	0	0				
Alcohol consumption *	No	27	54	33	66	46	62	18.203	<0.001
	Yes	23	46	17	34	4	8		

* Maximum two standard drinks a week. P; Patient Group, SC; Smoking Control Group, NSC; Non-smoking Control Group

**Table 2:** Comparison of psychic experiences scale data of patient and control groups.

	P (*n* = 50)	SC (*n* = 50)	NSC (*n* = 50)		
Variables	Mean ± SD	Mean ± SD	Mean ± SD	Statistic	*p*
Positive	30.16 ± 7.56	24.98 ± 3.78	25.66 ± 5.08	χ^2^ = 18.75	<0.001
Negative	24.16 ± 5.86	22.20 ± 4.96	24.10 ± 5.69	F = 2.36	0.134
Depressive	15.16 ± 4.15	12.04 ± 2.97	12.88 ± 3.06	χ^2^ = 15.50	<0.001
Total	69.48 ± 15.78	59.22 ± 10.17	62.64 ± 11.39	F = 8.49	<0.001
Distress level	65.42 ± 14.74	55.02 ± 9.88	55.84 ± 10.33	F = 11.89	<0.001

P; Patient Group, SC; Smoking Control Group, NSC; Non-smoking Control Group

**Table 3:** Comparison of DAT (SLC6A3) and VMAT2 (SLC18A2) gene expression and protein levels of patient and control groups.

	Variables	Group	*n*	Mean	SD	F	*p*
*DAT*	mRNA (*SLC6A3*)	P	46	2.74	1.30	107.003	<0.001 *
		SC	33	3.65	1.92		
		NSC	31	0.44	0.27		
	Protein	P	44	27.84	15.16	4.525	0.015 *
		SC	33	34.38	18.78		
		NSC	32	47.83	38.25		
*VMAT2*	mRNA (*SLC18A2*)	P	42	0.46	0.29	17.345	<0.001 *
		SC	32	1.18	0.78		
		NSC	31	1.02	0.79		
	Protein	P	44	14.79	10.19	3.283	0.044
		SC	30	13.23	8.51		
		NSC	31	21.71	16.18		

* (*p* < 0.017). P; Patient Group, SC; Smoking Control Group, NSC; Non-smoking Control Group

**Table 4:** Correlation of gene expression and protein levels with sociodemographic data in control groups.

		*SLC6A3* *(DAT)*		*SLC18A2* *(VMAT2)*		DAT (ELISA)		VMAT-2 (ELISA)	
		SC *n* = 33	NSC *n* = 31	SC *n* = 33	NSC *n* = 31	SC *n* = 33	NSC *n* = 31	SC *n* = 33	NSC *n* = 31
Age	r *p*	−0.109 0.545	−0.165 0.375	−0.071 0.701	−0.218 0.238	−0.248 0.163	0.092 0.616	−0.051 0.789	0.033 0.860
BMI	r *p*	0.046 0.798	−0.074 0.691	−0.323 0.072	−0.040 0.829	0.020 0.910	−0.018 0.922	0.019 0.923	−0.041 0825
First smoking age	r *p*	0.166 0.355	0.234 0.489	0.038 0.835	−0.341 0.278	0.143 0.428	−0.098 0.761	0.107 0.573	−0.161 0.636
Education level	r_s_ *p*	0.101 0.574	0.180 0.332	−0.122 0.504	0.150 0.420	−0.058 0.749	0.091 0.620	0.110 0.562	0.222 0.231
Monthly income	r_s_ *p*	0.237 0.185	−0.195 0.292	0.159 0.385	0.005 0.978	−0.038 0.833	0.372 0.036 *	0.130 0.495	0.355 0.050 *
Caffeine consumption	r_s_ *p*	−0.045 0.806	−0.380 0.035 *	−0.164 0.369	−0.585 0.001 **	0.126 0.484	0.156 0.393	0.023 0.905	0.223 0.229
Physical activity	r_s_ *p*	0.019 0.915	−0.007 0.969	0.100 0.586	−0.134 0.474	−0.290 0.102	0.064 0.726	−0.354 0.055	0.231 0.211

**. *p* < 0.01, *. *p* < 0.05) r: Pearson correlation coefficient r_s_: Spearman correlation coefficient, SC; Smoking Control Group, NSC; Non-smoking Control Group.

**Table 5:** A multinomial regression model was constructed with the SC group designated as the reference category.

Groups		B	SS	Wald	*p*	Exp(B)	95% CI	
							Lower	Upper
P	**Intercept**	4.769	1.235	14.925	0.000			
	**DAT**	−0.070	0.032	4.806	0.028	0.933	0.876	0.993
	**VMAT2**	0.112	0.067	2.789	0.095	1.119	0.981	1.277
	***SLC6A3***	−0.535	0.244	4.802	0.028	0.586	0.363	0.945
	***SLC18A2***	−2.895	0.901	10.329	0.001	0.055	0.009	0.323
NSC	**İntercept**	9.042	2.858	10.011	0.002			
	**DAT**	−0.111	0.068	2.710	0.100	0.895	0.784	1.021
	**VMAT2**	0.281	0.170	2.750	0.097	1.325	0.950	1.847
	***SLC6A3***	−12.645	5.099	6.149	0.013	0.00000322	1.471	0.071
	***SLC18A2***	4.261	3.168	1.809	0.179	70.895	0.143	35,267.154

**SC;** DAT (B;0.181, Exp (B); 1.20), VMAT-2 (B; −0.393, Exp (B); 0.675), ***SLC6A3*** (B;13.180 Exp (B); 529665), ***SLC18A2***(B; −1.366, Exp (B); 0.255), P; Patient Group, SC; Smoking Control Group, NSC; Non-smoking Control Group.

**Table 6:** A multinomial regression model was constructed with the NSC group designated as the reference category.

Groups		B	SS	Wald	*p*	Exp(B)	95% CI	
							Lower	Upper
P	Intercept	**−4.272**	**2.719**	**2.469**	**0.116**			
	DAT	0.041	0.066	0.394	0.530	1.042	0.916	1.186
	VMAT-2	−0.169	0.160	1.109	0.292	0.845	0.617	1.156
	*SLC6A3*	12.110	5.092	5.656	**0.017**	181,761	8.413	392,672
	*SLC18A2*	−7.156	3.231	4.907	**0.027**	0.001	0.000	0.439
SC	Intercept	−9.042	2.858	10.011	0.002			
	DAT	0.111	0.068	2.710	0.100	1.118	0.979	1.276
	VMAT-2	−0.281	0.170	2.750	0.097	0.755	0.542	1.052
	*SLC6A3*	12.645	5.099	6.149	**0.013**	310,352	14.165	679,987
	*SLC18A2*	−4.261	3.168	1.809	0.179	0.014	0.000	7.017

**NSC**; DAT (B; −0.152, Exp (B); 0.86) VMAT-2 ((B; 0.45, Exp (B); 1.57), *SLC6A3* (B; −24.755 Exp (B); 1.77e-11), *SLC18A2*(B;11.417 Exp (B); 90853), P; Patient Group, SC; Smoking Control Group, NSC; Non-smoking Control Group.

## Data Availability

The datasets used and analysed during the current study are available from the corresponding author at reasonable request.

## References

[1] Courtney K.E., Ray L.A. (2014). Methamphetamine: An update on epidemiology, pharmacology, clinical phenomenology, and treatment literature. Drug Alcohol Depend..

[2] European Monitoring Centre for Drugs and Drug Addiction (2022). European Drug Report 2022: Trends and Developments.

[3] Cruickshank C.C., Dyer K.R. (2009). A review of the clinical pharmacology of methamphetamine. Addiction.

[4] Iyo M., Namba H., Yanagisawa M., Hirai S., Yui N., Fukui S. (1997). Abnormal cerebral perfusion in chronic methamphetamine abusers: A study using 99MTc-HMPAO and SPECT. Prog. Neuro-Psychopharmacol. Biol. Psychiatry.

[5] Fiorentini A., Cantù F., Crisanti C., Cereda G., Oldani L., Brambilla P. (2021). Substance-Induced Psychoses: An Updated Literature Review. Front. Psychiatry.

[6] Gicas K.M., Parmar P.K., Fabiano G.F., Mashhadi F. (2022). Substance-induced psychosis and cognitive functioning: A systematic review. Psychiatry Res..

[7] Arunogiri S., McKetin R., Verdejo-Garcia A., Lubman D.I. (2020). The Methamphetamine-Associated Psychosis Spectrum: A Clinically Focused Review. Int. J. Ment. Health Addict..

[8] McHugh P.C., Buckley D.A. (2015). Chapter Eleven—The Structure and Function of the Dopamine Transporter and its Role in CNS Diseases. Vitamins & Hormones, Litwack, G., Ed..

[9] Amenta F., Bronzetti E., Cantalamessa F., El-Assouad D., Felici L., Ricci A., Tayebati S.K. (2001). Identification of dopamine plasma membrane and vesicular transporters in human peripheral blood lymphocytes. J. Neuroimmunol..

[10] Mill J., Asherson P., Browes C., D’Souza U., Craig I. (2002). Expression of the dopamine transporter gene is regulated by the 3’ UTR VNTR: Evidence from brain and lymphocytes using quantitative RT-PCR. Am. J. Med. Genet..

[11] Salatino-Oliveira A., Rohde L.A., Hutz M.H. (2018). The dopamine transporter role in psychiatric phenotypes. Am. J. Med. Genetics. Part B Neuropsychiatr. Genet. Off. Publ. Int. Soc. Psychiatr. Genet..

[12] Zahniser N.R., Sorkin A. (2004). Rapid regulation of the dopamine transporter: Role in stimulant addiction?. Neuropharmacology.

[13] Kordi-Tamandani D.M., Tajoddini S., Salimi F. (2015). Promoter Methylation and BDNF and DAT1 Gene Expression Profiles in Patients with Drug Addiction. Pathobiol. J. Immunopathol. Mol. Cell. Biol..

[14] O’Neill C.E., Levis S.C., Schreiner D.C., Amat J., Maier S.F., Bachtell R.K. (2015). Effects of adolescent caffeine consumption on cocaine sensitivity. Neuropsychopharmacol. Off. Publ. Am. Coll. Neuropsychopharmacol..

[15] Fagerberg L., Hallström B.M., Oksvold P., Kampf C., Djureinovic D., Odeberg J., Habuka M., Tahmasebpoor S., Danielsson A., Edlund K. (2014). Analysis of the human tissue-specific expression by genome-wide integration of transcriptomics and antibody-based proteomics. Mol. Cell. Proteom. MCP.

[16] van Os J., Linscott R.J., Myin-Germeys I., Delespaul P., Krabbendam L. (2009). A systematic review and meta-analysis of the psychosis continuum: Evidence for a psychosis proneness-persistence-impairment model of psychotic disorder. Psychol. Med..

[17] Chiang M., Lombardi D., Du J., Makrum U., Sitthichai R., Harrington A., Shukair N., Zhao M., Fan X. (2019). Methamphetamine-associated psychosis: Clinical presentation, biological basis, and treatment options. Hum. Psychopharmacol..

[18] Kalayasiri R., Kraijak K., Maes M., Mutirangura A. (2019). Methamphetamine (MA) Use Induces Specific Changes in LINE-1 Partial Methylation Patterns, Which Are Associated with MA-Induced Paranoia: A Multivariate and Neuronal Network Study. Mol. Neurobiol..

[19] Boileau I., Rusjan P., Houle S., Wilkins D., Tong J., Selby P., Guttman M., Saint-Cyr J.A., Wilson A.A., Kish S.J. (2008). Increased vesicular monoamine transporter binding during early abstinence in human methamphetamine users: Is VMAT2 a stable dopamine neuron biomarker?. J. Neurosci. Off. J. Soc. Neurosci..

[20] Guydish J., Le T., Hosakote S., Straus E., Wong J., Martínez C., Delucchi K. (2022). Tobacco use among substance use disorder (SUD) treatment staff is associated with tobacco-related services received by clients. J. Subst. Abus. Treat..

[21] Zhu J., Reith M.E. (2008). Role of the dopamine transporter in the action of psychostimulants, nicotine, and other drugs of abuse. CNS Neurol. Disord. Drug Targets.

[22] López A.J., Siciliano C.A., Calipari E.S. (2020). Activity-Dependent Epigenetic Remodeling in Cocaine Use Disorder. Handb. Exp. Pharmacol..

[23] Werner C.T., Altshuler R.D., Shaham Y., Li X. (2021). Epigenetic Mechanisms in Drug Relapse. Biol. Psychiatry.

[24] Bimpisidis Z., Serra G.P., König N., Wallén-Mackenzie Å. (2023). Increased sucrose consumption in mice gene-targeted for Vmat2 selectively in NeuroD6-positive neurons of the ventral tegmental area. Front. Mol. Neurosci..

[25] Balci F., Ludvig E.A., Abner R., Zhuang X., Poon P., Brunner D. (2010). Motivational effects on interval timing in dopamine transporter (DAT) knockdown mice. Brain Res..

[26] Bristow G.C., Eisenlohr-Moul T., Lotesto K., Sodhi M.S. (2021). Sex differences in the transcription of monoamine transporters in major depression. J. Affect. Disord..

[27] Yazici A.B., Yazici E., Akkisi Kumsar N., Erol A. (2015). Addiction profile in probation practices in Turkey: 5-year data analysis. Neuropsychiatr. Dis. Treat..

[28] Faul F., Erdfelder E., Lang A.-G., Buchner A. (2007). G* Power 3: A flexible statistical power analysis program for the social, behavioral, and biomedical sciences. Behav. Res. Methods.

[29] Ögel K., Evren C., Karadağ F., Gürol T. (2012). Bağımlılık Profil İndeksi’nin (BAPİ) geliştirilmesi, geçerlik ve güvenilirliği. Türk Psikiyatr. Derg..

[30] Stefanis N., Hanssen M., Smirnis N., Avramopoulos D., Evdokimidis I., Stefanis C., Verdoux H., van Os J. (2002). Evidence that three dimensions of psychosis have a distribution in the general population. Psychol. Med..

[31] Sevi O.M., Ustamehmetoğlu F., Gülen M., Zeybek Z. (2019). The Reliability and Validity of Community Assessment of Psychic Experiences Scale-Turkish Form. Yeni Symp..

[32] Evren C., Saatçioğlu Ö., Dalbudak E., Danışmant B.S., Çakmak D., Ryan R.M. (2006). Tedavi motivasyonu anketi (TMA) Türkçe versiyonunun alkol bağımlısı hastalarda faktör yapısı, geçerliği ve güvenirliği. Bağımlılık Derg..

[33] Ryan R.M., Plant R.W., O’Malley S. (1995). Initial motivations for alcohol treatment: Relations with patient characteristics, treatment involvement, and dropout. Addict. Behav..

[34] Turan Ç., Budak E., Şenormancı G., Evren C., Ünal S., Yalçınkaya E.A., Senormanci Ö. (2023). Risk of Relapse Assessment Scale for Metamphetamine Abusers: Reliability and Validity Study of the Turkish Version. Psychiatry Clin. Psychopharmacol..

[35] Evren C., Dalbudak E., Çakmak D. (2008). Değişime Hazır Olma ve Tedavi İsteği Ölçeği (SOCRATES) Türkçe Versiyonu’nun Yatarak Tedavi Gören Erkek Alkol Bağımlısı Hastalarda Faktör Yapısı, Geçerliliği ve Güvenirliği. Klin. Psikofarmakol. Bülteni.

[36] George D., Mallery P. (2019). IBM SPSS Statistics 26 Step by Step: A Simple Guide and Reference.

[37] Pallant J. (2017). SPSS Kullanma Kılavuzu. BA Sibel Balcı, Çev.).

[38] Diedenhofen B., Musch J. (2015). cocor: A comprehensive solution for the statistical comparison of correlations. PLoS ONE.

[39] TUBIM (2022). Madde Kullanicilari Profil Analizi 2021 Yili Narkolog Raporu. https://www.narkotik.pol.tr/kurumlar/narkotik.pol.tr/TUB%C4%B0M/Ulusal%20Yay%C4%B1nlar/NARKOLOG-2023-PROFIL-ANALIZI.pdf.

[40] Ji J., Bourque M., Di Paolo T., Dluzen D.E. (2009). Genetic alteration in the dopamine transporter differentially affects male and female nigrostriatal transporter systems. Biochem. Pharmacol..

[41] Biederman J., Petty C.R., Hammerness P., Batchelder H., Faraone S.V. (2018). Cigarette smoking as a risk factor for other substance misuse: 10-year study of individuals with and without attention-deficit hyperactivity disorder. Br. J. Psychiatry.

[42] Erdoğan Kaya A., Yazici A.B., Kaya M., Yazici E. (2021). The relationship between expressed emotion, personality traits and prognosis of alcohol and substance addiction: 6-month follow-up study. Nord. J. Psychiatry.

[43] Rognli E.B., Bramness J.G., Skurtveit S., Bukten A. (2017). Substance use and sociodemographic background as risk factors for lifetime psychotic experiences in a non-clinical sample. J. Subst. Abus. Treat..

[44] Matheson S.L., Laurie M., Laurens K.R. (2023). Substance use and psychotic-like experiences in young people: A systematic review and meta-analysis. Psychol. Med..

[45] Degenhardt L., Saha S., Lim C.C.W., Aguilar-Gaxiola S., Al-Hamzawi A., Alonso J., Andrade L.H., Bromet E.J., Bruffaerts R., Caldas-de-Almeida J.M. (2018). The associations between psychotic experiences and substance use and substance use disorders: Findings from the World Health Organization World Mental Health surveys. Addiction.

[46] Murali V., Oyebode F. (2018). Poverty, social inequality and mental health. Adv. Psychiatr. Treat..

[47] Giannouli V. (2017). Violence in severe mental illness: Is cognition missing in the associations with ethnicity, cannabis and alcohol?. Australas. Psychiatry.

[48] Lapworth K., Dawe S., Davis P., Kavanagh D., Young R., Saunders J. (2009). Impulsivity and positive psychotic symptoms influence hostility in methamphetamine users. Addict. Behav..

[49] Gage S.H., Munafò M.R. (2015). Rethinking the association between smoking and schizophrenia. Lancet Psychiatry.

[50] Saha S., Scott J.G., Varghese D., Degenhardt L., Slade T., McGrath J.J. (2011). The association between delusional-like experiences, and tobacco, alcohol or cannabis use: A nationwide population-based survey. BMC Psychiatry.

[51] Bhavsar V., Jauhar S., Murray R.M., Hotopf M., Hatch S.L., McNeill A., Boydell J., MacCabe J.H. (2018). Tobacco smoking is associated with psychotic experiences in the general population of South London. Psychol. Med..

[52] Volz T.J., Fleckenstein A.E., Hanson G.R. (2007). Methamphetamine-induced alterations in monoamine transport: Implications for neurotoxicity, neuroprotection and treatment. Addiction.

[53] Staley J.K., Krishnan-Sarin S., Zoghbi S., Tamagnan G., Fujita M., Seibyl J.P., Maciejewski P.K., O’Malley S., Innis R.B. (2001). Sex differences in [123I] β-CIT SPECT measures of dopamine and serotonin transporter availability in healthy smokers and nonsmokers. Synapse.

[54] Leroy C., Karila L., Martinot J.L., Lukasiewicz M., Duchesnay E., Comtat C., Dollé F., Benyamina A., Artiges E., Ribeiro M.J. (2012). Striatal and extrastriatal dopamine transporter in cannabis and tobacco addiction: A high-resolution PET study. Addict. Biol..

[55] Newberg A., Lerman C., Wintering N., Ploessl K., Mozley P.D. (2007). Dopamine transporter binding in smokers and nonsmokers. Clin. Nucl. Med..

[56] Grzywacz A., Barczak W., Chmielowiec J., Chmielowiec K., Suchanecka A., Trybek G., Masiak J., Jagielski P., Grocholewicz K., Rubiś B. (2020). Contribution of dopamine transporter gene methylation status to cannabis dependency. Brain Sci..

[57] Chen Y., Zhang J., Sun Y. (2019). The relationship between childhood abuse and depression in a sample of Chinese people who use methamphetamine. Int. J. Clin. Health Psychol..

[58] Zhou Y., Michelhaugh S.K., Schmidt C.J., Liu J.S., Bannon M.J., Lin Z. (2014). Ventral midbrain correlation between genetic variation and expression of the dopamine transporter gene in cocaine-abusing versus non-abusing subjects. Addict. Biol..

[59] Imam S.Z., Duhart H.M., Skinner J.T., Ali S.F. (2005). Cocaine induces a differential dose-dependent alteration in the expression profile of immediate early genes, transcription factors, and caspases in PC12 cells: A possible mechanism of neurotoxic damage in cocaine addiction. Ann. N. Y. Acad. Sci..

[60] Yamashita H., Kitayama S., Zhang Y.-X., Takahashi T., Dohi T., Nakamura S. (1995). Effect of nicotine on dopamine uptake in COS cells possessing the rat dopamine transporter and in PC12 cells. Biochem. Pharmacol..

[61] Zhu J., Bruntz R.C., Apparsundaram S., Dwoskin L.P. (2009). Nicotinic receptor activation increases [H-3] dopamine uptake and cell surface expression of dopamine transporters in rat prefrontal cortex. J. Pharmacol. Exp. Ther..

[62] Li S., Kim K.Y., Kim J.H., Kim J.H., Park M.S., Bahk J.Y., Kim M.O. (2004). Chronic nicotine and smoking treatment increases dopamine transporter mRNA expression in the rat midbrain. Neurosci. Lett..

[63] Kitamura O. (2009). Detection of methamphetamine neurotoxicity in forensic autopsy cases. Leg Med..

[64] Brown J.M., Riddle E.L., Sandoval V., Weston R.K., Hanson J.E., Crosby M.J., Ugarte Y.V., Gibb J.W., Hanson G.R., Fleckenstein A.E. (2002). A single methamphetamine administration rapidly decreases vesicular dopamine uptake. J. Pharmacol. Exp. Ther..

[65] Ugarte Y.V., Rau K.S., Riddle E.L., Hanson G.R., Fleckenstein A.E. (2003). Methamphetamine rapidly decreases mouse vesicular dopamine uptake: Role of hyperthermia and dopamine D2 receptors. Eur. J. Pharmacol..

[66] Duchemin A.-M., Zhang H., Neff N.H., Hadjiconstantinou M. (2009). Increased expression of VMAT2 in dopaminergic neurons during nicotine withdrawal. Neurosci. Lett..

[67] Eyerman D.J., Yamamoto B.K. (2007). A rapid oxidation and persistent decrease in the vesicular monoamine transporter 2 after methamphetamine. J. Neurochem..

[68] Fleckenstein A.E., Volz T.J., Hanson G.R. (2009). Psychostimulant-induced alterations in vesicular monoamine transporter-2 function: Neurotoxic and therapeutic implications. Neuropharmacology.

[69] Miner N.B., Phillips T.J., Janowsky A. (2019). The Role of Biogenic Amine Transporters in Trace Amine-Associated Receptor 1 Regulation of Methamphetamine-Induced Neurotoxicity. J. Pharmacol. Exp. Ther..

[70] Lohr K.M., Stout K.A., Dunn A.R., Wang M., Salahpour A., Guillot T.S., Miller G.W. (2015). Increased Vesicular Monoamine Transporter 2 (VMAT2; Slc18a2) Protects against Methamphetamine Toxicity. ACS Chem. Neurosci..

[71] Guillot T.S., Richardson J.R., Wang M.Z., Li Y.J., Taylor T.N., Ciliax B.J., Zachrisson O., Mercer A., Miller G.W. (2008). PACAP38 increases vesicular monoamine transporter 2 (VMAT2) expression and attenuates methamphetamine toxicity. Neuropeptides.

[72] Diez G.G., Martin-Subero I., Zangri R.M., Kulis M., Andreu C., Blanco I., Roca P., Cuesta P., García C., Garzón J. (2023). Epigenetic, psychological, and EEG changes after a 1-week retreat based on mindfulness and compassion for stress reduction in healthy adults: Study protocol of a cross-over randomized controlled trial. PLoS ONE.

